# Brain lipidomics for region-specific biomarker discovery in neurodegenerative diseases

**DOI:** 10.3389/fnagi.2026.1757306

**Published:** 2026-02-11

**Authors:** Jayashankar Jayaprakash, Solomon Tebeje Gizaw, Divyavani Gowda, Hiroshi Hinou, Shin-Ichiro Nishimura, Shu-Ping Hui, Siddabasave Gowda B. Gowda

**Affiliations:** 1 Graduate School of Global Food Resources, Hokkaido University, Sapporo, Japan; 2 Department of Medical Biochemistry, School of Medicine, College of Health Sciences, Addis Ababa University, Addis Ababa, Ethiopia; 3 Faculty of Health Sciences, Hokkaido University, Sapporo, Japan; 4 Frontier Research Center for Advanced Material and Life Science, Faculty of Advanced Life Science, Hokkaido University, Sapporo, Japan

**Keywords:** Alzheimer’s disease, Huntington’s disease, lipid biomarkers, lipidomics, liquid chromatography, mass spectrometry, Parkinson’s disease

## Abstract

**Background:**

Alzheimer’s disease (AD), Parkinson’s disease (PD), and Huntington’s disease (HD) are progressive neurodegenerative diseases (NDs) characterized by chronic neuronal loss. The lack of effective treatments highlights the urgent need for reliable lipid biomarkers to enable diagnosis and monitor disease progression. Previous lipidomic investigations of altered lipid metabolism have focused on a single disease type, limiting cross-disease comparisons.

**Methods:**

We applied the untargeted liquid chromatography-mass spectrometry (LC/MS) technique to profile brain lipidome alterations and to identify disease-specific lipid biomarkers across AD, HD, and PD. Brain tissue samples were collected from four cerebral lobes of healthy volunteers (HV, *n* = 24) and patients diagnosed with AD (*n* = 24), PD (*n* = 24), and HD (*n* = 24). All groups include three males and three females, with brain tissues from four cortical regions sacrificed from each individual.

**Results:**

A total of 243 lipid molecular species spanning five major classes were annotated, revealing distinct disease-specific lipidomic profiles that differentiated HV from the AD, HD, and PD groups via multivariate analysis. Sphingomyelins and oxidized phosphatidylserine [PS (16:1/24:0;O1)] were significantly increased, while lysophosphatidylcholines (LPC 18:2, LPC 17:2) were decreased in the AD group relative to HV. HD exhibited elevated PS (O-17:0/22:6) and ω-6 fatty acid esterified cholesteryl esters (CE 18:2, CE 20:4), alongside decreased essential neuronal lipids such as phosphatidylinositols (PI). The PD lipidome alterations closely resembled those of HD, indicating partially overlapping disruptions in brain lipid metabolism. Receiver operating characteristic analysis identified PS (16:1/24:0;O1), PS (O-17:0/22:6), and PI (18:1/18:1) as potential discriminatory biomarkers with strong diagnostic performance. Regional heatmap analysis revealed significant lipid perturbations were observed in the parietal and occipital lobes across all NDs.

**Conclusion:**

This study provides a comprehensive overview of disease- and region-specific alterations in the brain lipidome of AD, HD, and PD. The identified lipid species-PS (16:1/24:0;O1), PS (O-17:0/22:6), and PI (18:1/18:1)-may serve as promising candidate biomarkers for NDs diagnosis and warrant further mechanistic and longitudinal validation with large data set.

## 1 Introduction

Neurodegenerative diseases (NDs) are chronic and progressive disorders characterized by the selective loss of neurons and associated cellular connections, leading to motor, cognitive, and behavioral impairments (Lamptey et al., 2022). Among the most prevalent NDs are Alzheimer’s disease (AD), Parkinson’s disease (PD), and Huntington’s disease (HD), which collectively impose a substantial socio-economic burden on the aging population worldwide (GBD 2016 Parkinson’s Disease Collaborators, 2018; GBD 2019 Dementia Forecasting Collaborators, 2022). AD, the most common cause of dementia in older adults, is marked by progressive memory loss and cognitive decline, accompanied by the accumulation of phosphorylated tau (p-tau) and β-amyloid (Aβ) proteins (Bloom, 2014). PD, the second most common ND after AD, is characterized by degeneration of dopaminergic neurons in the substantia nigra, leading to α-synuclein aggregation and Lewy body formation that contribute to both motor and non-motor symptoms (Rissardo et al., 2025). HD, in contrast, results from abnormal expansion of the cytosine-adenine guanine (CAG) trinucleotide repeat in the Huntington gene, producing a mutant huntingtin protein that drives neuronal degeneration in the striatum and cortex (Yilmaz et al., 2025a). These disorders typically manifest in mid-to-late life and are often preceded by long preclinical phases during which molecular alterations occur before clinical symptoms appear (Katsuno et al., 2018). Despite major research advances, accurate diagnosis of NDs remains challenging, emphasizing the urgent need for reliable and disease-specific biomarkers (Khoury and Ghossoub, 2019; Li and Le, 2020).

Current diagnostic approaches-such as invasive sampling, fluid-based protein assays, and neuroimaging techniques lack specificity for NDs detection and diagnosis (Hansson, 2021; Selvam and Ayyavoo, 2024). Lipidomic profiling offers distinct advantages by enabling comprehensive characterization of lipid alterations and identification of novel lipid biomarkers associated with disease onset and progression (Zhang et al., 2020). As lipids constitute ~50% of the brain’s dry weight, their balanced metabolism is essential for maintaining cell signaling, proliferation, apoptosis, and neurotransmission (O’Brien and Sampson, 1965; Hornemann, 2021). Disruptions in lipid homeostasis have been closely linked to NDs progression (Martens et al., 2023; Feringa et al., 2025), positioning brain lipids as promising candidates for potential diagnostic and prognostic biomarkers (Yoon et al., 2022). Previous liquid chromatography/mass spectrometry (LC/MS)-based lipidomic analyses have revealed elevated levels of glycerophospholipids (GPs) and sphingolipids (SPs) in AD brain tissue (Sanni et al., 2025), as well as alterations in triacylglycerol (TG) and lysophosphatidylcholine (LPC) levels in PD and changes in diacylglycerol and monoacylglycerol levels in HD (Paryani et al., 2024; Yilmaz et al., 2025b). However, these studies largely focused on single neurological conditions, and most were conducted using plasma or cerebrospinal fluid (CSF) samples, limiting insights into region-specific lipidomic alterations within the brain (Byeon et al., 2021; Liu et al., 2021).

**Figure 1:**
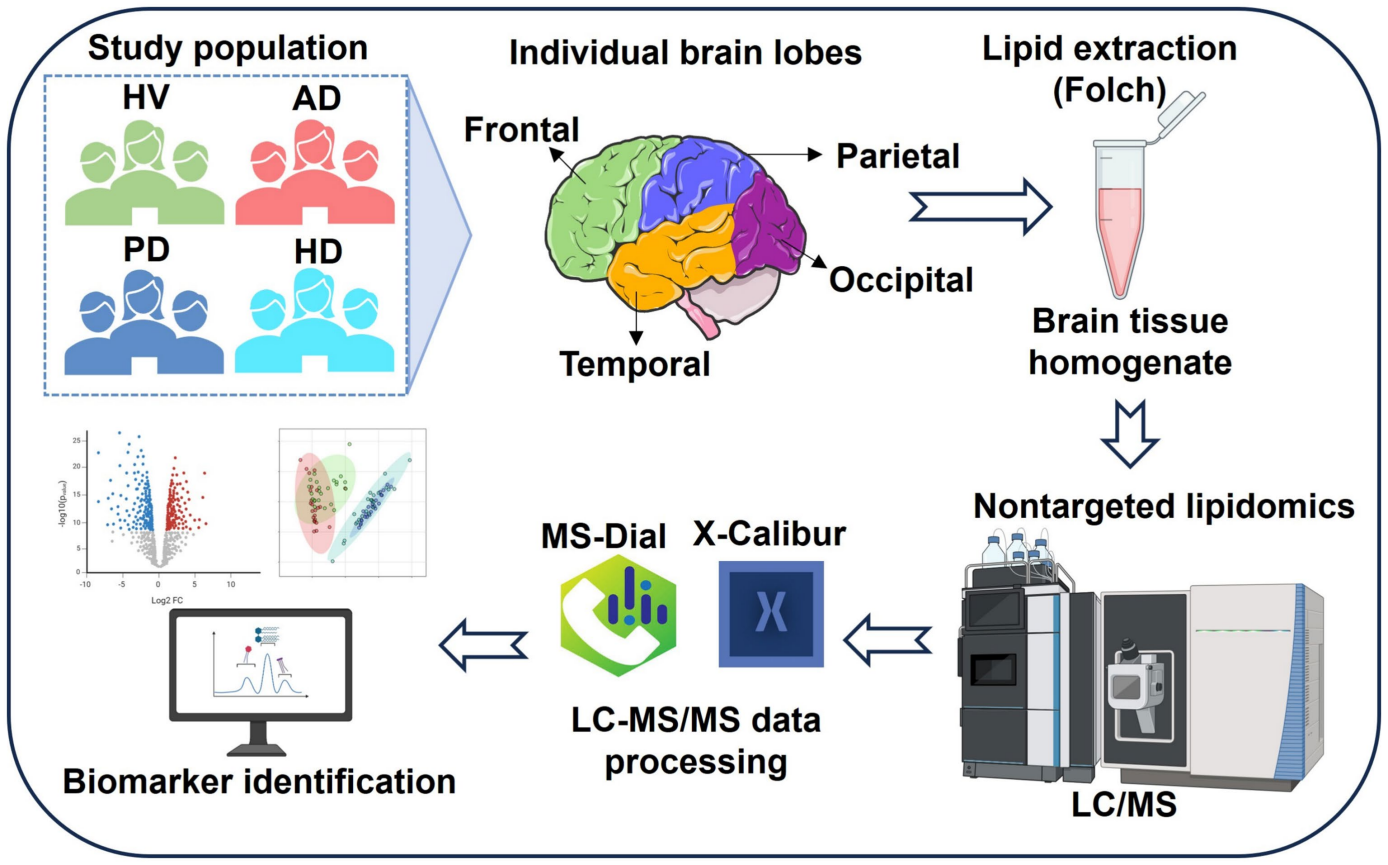
Study design for lipidomic analysis of brain tissue samples collected from healthy volunteers (HV) and individuals with Alzheimer’s disease (AD), Parkinson’s disease (PD), and Huntington’s disease (HD). Sample sizes: HV (*n* = 24), AD (*n* = 24), PD (*n* = 24), and HD (*n* = 24).

To address these limitations, the present study employed high-performance liquid chromatography coupled with linear trap quadrupole-Orbitrap mass spectrometry (HPLC/LTQ-Orbitrap-MS) to perform a comprehensive lipidomic analysis of brain tissue samples from healthy volunteers (HV) and patients with AD, HD, and PD. The study further examined sex- and region-specific lipid variations, as summarized in Figure 1.

## 2 Materials and methods

### 2.1 Chemicals

High-purity LC/MS-grade solvents, including isopropanol, chloroform, and methanol, were obtained from Wako Pure Chemical Industries, Ltd. (Osaka, Japan). A 1 M aqueous solution of ammonium acetate, used as an additive in the LC/MS mobile phase, was purchased from Sigma-Aldrich (St. Louis, MO, United States). Oleic acid-d9 and EquiSPLASH LIPIDOMIX quantitative standard mixture (Lot: 330731-1EA-013) were procured from Avanti Polar Lipids (Alabaster, AL, United States).

### 2.2 Sample information

Human brain tissue samples from four cerebral lobes were obtained from healthy volunteers (HV, *n* = 24) and patients diagnosed with Alzheimer’s disease (AD, *n* = 24), Parkinson’s disease (PD, *n* = 24), and Huntington’s disease (HD, *n* = 24). The samples were provided by the Human Brain and Spinal Fluid Resource Center (HBSFRC), VA West Los Angeles Healthcare Center, Los Angeles, CA 90073, supported by the National Institute of Neurological Disorders and Stroke (NINDS)/National Institute of Mental Health (NIMH), and the National Multiple Sclerosis Society, United States. Ethical approval for this study was obtained from the Institutional Review Board of the Graduate School of Advanced Life Sciences, Hokkaido University (Approval No. 07-01). Four cortical regions—frontal, temporal, parietal, and occipital-were dissected from 24 male and female subjects (a total of 96 cerebral lobes), following previously described protocols (Gizaw, 2015), and stored at −80 °C until analysis. Detailed demographic and specimen characteristics, including brain region, sex, autolysis time, and age, are presented in Table 1.

**Table 1:** Demographic and clinical characteristics of study participants.

Condition	Tissue samples of the cerebral cortices (*n* = 3, all cases)				Age (average years ± SD)	Autolysis time (hours ± SD)
	Frontal	Temporal	Parietal	Occipital		
Healthy volunteer (HV)	Male				66 ± 9.5	15.7 ± 5.6
	Female				67 ± 11	17.8 ± 2.2
Alzheimer’s disease (AD)	Male				77 ± 2.8	13.1 ± 3.0
	Female				63 ± 16.7	17.0 ± 1.9
Parkinson’s disease (PD)	Male				80 ± 7	18.2 ± 10.7
	Female				72 ± 8.5	15.2 ± 0.83
Huntington’s disease (HD)	Male				54 ± 20.5	11.7 ± 4.0
	Female				72 ± 6.8	11.8 ± 3.7

SD, standard deviation.

### 2.3 Lipid extraction

Brain tissue samples from HV, AD, PD, and HD were subjected to total lipid extraction using a modified Folch method (Minami et al., 2025; Folch et al., 1957). In brief, 200 μL of brain tissue homogenate in PBS (2 mg/100 μL) was transferred to a 2 mL microcentrifuge tube, followed by the addition of 300 μL of ice-cold methanol containing 0.01% butylated hydroxytoluene and 100 μL of an internal standard (IS) solution consisting of oleic acid-d9 (10 μg/mL) and EquiSPLASH LIPIDOMIX mixtures in methanol (1 μg/mL). Subsequently, 800 μL of chloroform was added, and the mixture was vortexed for 5 min and centrifuged at 15,000 rpm for 10 min to achieve biphasic separation. The lower chloroform phase containing lipids was collected into a new 2 mL tube, and the aqueous phase was re-extracted with chloroform under the same conditions. The resulting chloroform extracts were combined and evaporated in a centrifugal evaporator at 4 °C for 3 h. The dried lipid residues were reconstituted in 100 μL of methanol, centrifuged at 15,000 rpm for 10 min, and the supernatants were analyzed by LC/MS with an injection volume of 20 μL per run. Simultaneously, blank and quality control (QC) samples (prepared by mixing multiple brain extracts) were acquired between the runs for data quality management. QC samples were employed to manage inter-batch variability throughout the analysis, and the limit of detection (LOD) for each lipid class was determined by analyzing internal standards prepared at different concentration levels. The determined LOD for the internal standards was in the range of 0.01 ng/mL to 1 ng/mL.

### 2.4 LC/MS analysis

Lipidomic analysis was performed using a high-performance liquid chromatography (HPLC) system (Shimadzu Corporation, Kyoto, Japan) coupled to an LTQ Orbitrap XL mass spectrometer (Thermo Fisher Scientific Inc., San Jose, CA, United States). Lipid separation was achieved on an Atlantis T3 C18 column (2.1 mm × 150 mm, 3 μm; Waters, Milford, MA, United States) maintained at 40 °C, with a flow rate of 0.2 mL/min. The mobile phase consisted of 10 mM aqueous ammonium acetate (A), isopropanol (B), and methanol (C), using an elution gradient identical to that described in our previous study (Nath et al., 2024). All mass spectrometric parameters were consistent with those reported previously (Jayaprakash et al., 2025). Data acquisition was performed in both positive and negative electrospray ionization (ESI) modes. In negative mode, the source and capillary voltages were set to 3 kV and 10 V, respectively, with a scan range of *m*/*z* 160–1,900. In positive mode, the corresponding voltages were 4 kV and 25 V, with a scan range of *m*/*z* 150–1,950. In both modes, the sheath and auxiliary nitrogen gas flow rates were 50 and 20 units, respectively, and the capillary temperature was maintained at 330 °C. Full MS spectra were acquired in Fourier transform mode at a resolving power of 60,000, while MS/MS spectra were obtained in ion trap mode using collision-induced dissociation (CID) at 40 V collision energy.

### 2.5 Lipid identification and quantification

Raw LC/MS data were processed using MS-DIAL software (version 4.9) for lipid peak integration and annotation. MS and MS/MS spectra were further examined in Xcalibur software (version 2.2; Thermo Fisher Scientific, Waltham, MA, United States) to confirm the accurate identification of lipid molecular species and to manually verify peak integration. The relative concentrations of lipid metabolites were determined semi-quantitatively based on the IS added during extraction. Data normalization was performed according to the sample weight used for analysis.

### 2.6 Statistical analysis

Data visualization was performed using Microsoft Excel 2021, and results were plotted with GraphPad Prism (version 8.0.1). To evaluate data variability, orthogonal partial least squares discriminant analysis (OPLS-DA) was conducted using MetaboAnalyst 6.0. OPLS-DA score plots were generated to assess group separation, and variable importance in projection (VIP) plots were used to identify key discriminatory lipid features. Statistical analyses were performed using ordinary two-way ANOVA followed by Bonferroni’s multiple comparison test for pairwise comparisons. Differences were considered statistically significant at *p* < 0.05. Data are presented as mean ± standard error of the mean (SEM).

## 3 Results

### 3.1 Multivariate analysis of the brain lipidome

**Figure 2:**
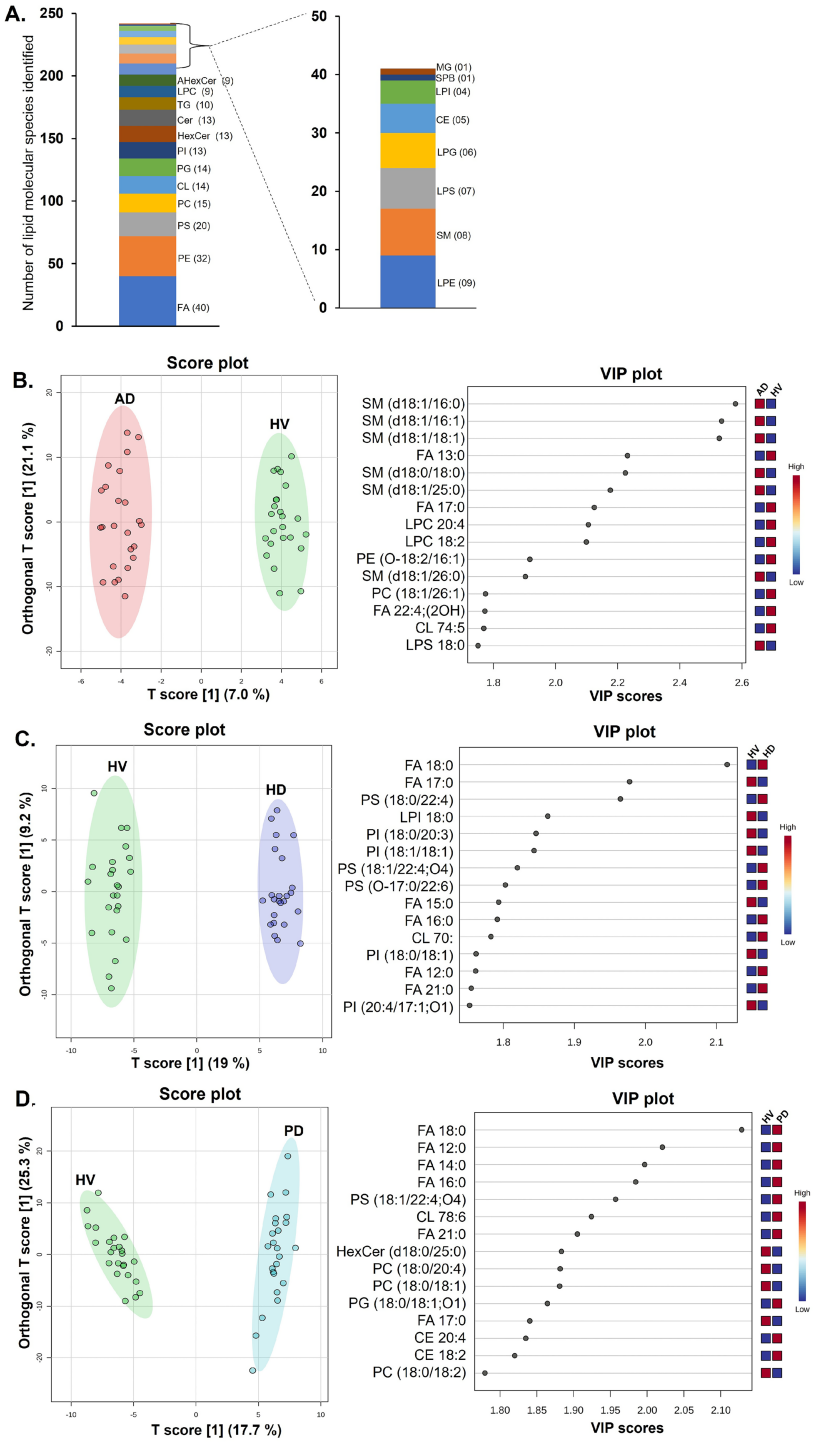
**(A)** Distribution of different lipid classes identified across brain regions of HV, AD, HD, and PD. Orthogonal partial least squares discriminant analysis (OPLS-DA) score plots and variable importance in projection (VIP) plots analysis for comparing lipidome profiles in **(B)** HV and AD groups, **(C)** HV and HD groups, **(D)** HV and PD groups.

Untargeted lipidomic profiling was performed on brain tissue samples obtained from the frontal, temporal, parietal, and occipital cortices of HV and patients diagnosed with AD, PD, and HD was conducted using HPLC/LTQ-Orbitrap-MS. The analysis identified 243 distinct lipid molecular species encompassing five major lipid classes, as shown in Figure 2A. Among these, 40 lipid metabolites were identified as fatty acyls (FAs), followed by 32 phosphatidylethanolamine (PEs), 20 phosphatidylserine (PSs), 15 phosphatidylcholine (PCs), 14 cardiolipin (CLs), 14 phosphatidylglycerol (PGs), 13 phosphatidylinositol (PIs), 13 ceramides (Cer), 13 hexosylceramides (HexCer), 10 TGs, 9 LPCs, 9 acyl-hexosylceramides (AHexCer), among others. The detailed list of each identified lipid molecular species, including their respective retention times and *m*/*z* values, is provided in Supplementary Table S1.

Multivariate statistical analyses were conducted to elucidate group- and disease-specific differences in brain lipid composition among the HV, AD, PD, and HD groups. OPLS-DA was applied to the lipid molecular profiles obtained from the four cortical regions, and the resulting score and VIP plots are presented in Figures 2B–D. The OPLS-DA score plot for HV and AD groups demonstrated clear separation, indicating distinct lipidomic signatures (Figure 2B). The first two components (T score 1 and T score 2) explained 28.1% of the total model variance. The corresponding VIP plot identified key lipid species contributing to group discrimination, including sphingolipids (e.g., SM d18:1/16:0), fatty acids (e.g., FA 13:0), and glycerophospholipids (e.g., LPC 20:4) with VIP scores greater than 2. Figure 2C illustrates the OPLS-DA score plot showing distinct separation between HV and HD groups, suggesting marked differences in lipid composition. The first two components accounted for 28.2% of the model variance. The associated VIP plot highlighted fatty acids (e.g., FA 18:0) and glycerophospholipids (e.g., PS 18:0/20:3) as major contributors to this separation. Similarly, as shown in Figure 2D, the OPLS-DA score plot revealed a clear group separation between HV and PD groups, with the model explaining 43% of the total variance, indicating pronounced lipidomic differences. The VIP plot highlighted key lipid species contributing to the group discrimination, belonging to fatty acids (e.g., FA 18:0), glycerophospholipids (PC 18:0/20:4), and sterols (CE 20:4).

### 3.2 Disease-specific alteration in brain lipidome and identification of biomarkers for AD, PD, and HD

**Figure 3:**
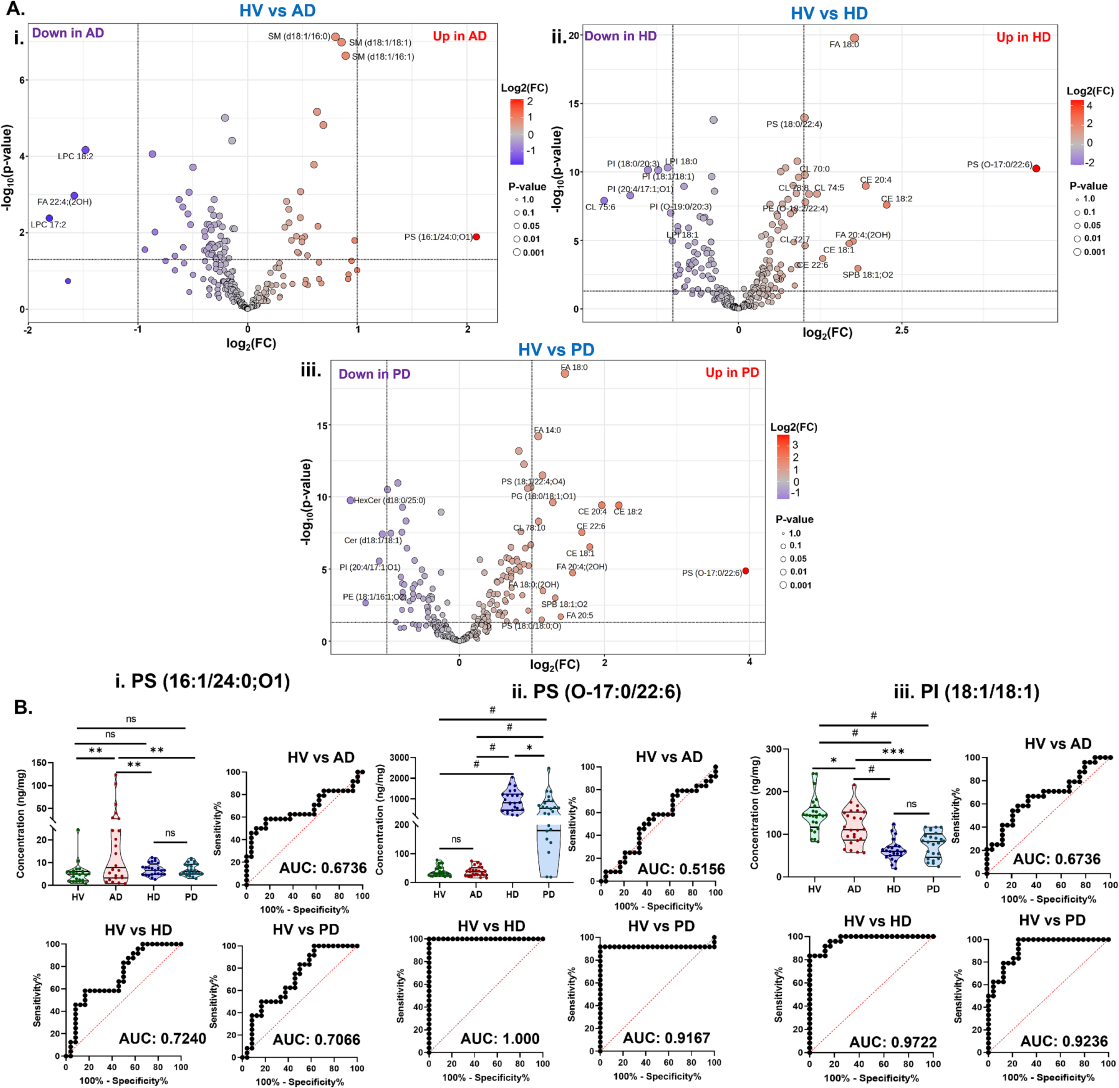
**(A)** Volcanic plot representing significantly altered lipids (unpaired *t*-test, *p* < 0.05) in brain tissue samples comparing **(i)** HV vs. AD, **(ii)** HV vs. HD, and **(iii)** HV vs. PD. **(B)** Violin plots and receiver operating characteristic (ROC) curves analysis for top discriminatory lipid markers: **(i)** PS (16:1/24:0;O1), **(ii)** PS (O-17:0/22:6), and **(iii)** PI (18:1/18:1) across AD, HD, and PD groups. [Ordinary two-way ANOVA with Bonferroni’s multiple comparisons test, (95% CI), ^#^*p* < 0.0001, ^***^p < 0.001, ^**^p < 0.01, ^*^p < 0.05, and 0.1(ns)]. All data are represented as mean ± SEM.

The volcano plots illustrate the distribution of significantly altered lipids between HV and disease groups (AD, HD, and PD) by plotting −log_10_(*p*-value) against log_2_(fold change), highlighting lipid species with both statistical significance and substantial fold changes (Figure 3A). Lipids with significantly higher abundance in disease groups appear in red on the right side of the plot, whereas those with decreased abundance are shown in blue on the left, reflecting disease-specific changes in lipid levels.

Compared with HV SM (d18:1/16:0), SM (d18:1/18:1), SM (d18:1/16:1), and PS (16:1/24:0;O1) were significantly increased in the AD groups. In contrast, LPC 17:2, LPC 18:2, and FA 22:4;(2OH) were significantly decreased relative to HV (Figure 3Ai). Comparison of HV and HD groups revealed an increase in several lipid species, including multiple cholesterol esters (CEs), cardiolipins (CLs), PS (18:0/22:4), FA 18:0, FA 20:4;(2OH), PE (O-18:2/22:4), and PS (O-17:0/22:6). Conversely, levels of phosphatidylinositol species-PI (18:0/20:3), PI (18:1/18:1), PI (O-19:0/20:3), PI (20:4/17:1;O1)-and lysophosphatidylinositol species LPI 18:0 and LPI 18:1 were decreased in HD compared with HV (Figure 3Aii). Similar lipidome alterations were observed in PD, with elevated levels of CEs, FAs, and other lipid species, such as PS (18:1/22:4;O4), CL 78:10, PG (18:0/18:1;O1), and PS (O-17:0/22:6). Decreased levels of HexCer (d18:0/25:0), Cer (d18:1/18:1), PI (20:4/17:1;O1), and PE (18:1/16:1;O2) were detected in PD relative to HV (Figure 3Aiii). A comprehensive list of the most significantly altered lipids and their fold-change values is provided in Supplementary Tables S2–S4.

Receiver operating characteristic (ROC) analysis was performed on lipid species exhibiting significant alterations between HV and disease cohorts to evaluate their potential as diagnostic biomarkers. Violin plots illustrating relative lipid concentrations across groups are presented alongside ROC curves to visualize discriminatory performance (Figure 3B). Among the significantly altered lipids in AD, PS (16:1/24:0;O1) showed notable diagnostic capability, yielding area under the curve (AUC) values of 0.6736 for AD, 0.7240 for HD, and 0.7066 for PD. The violin plot demonstrates a marked increase of this lipid in AD compared with HV, with non-significant differences in HD and PD (Figure 3Bi). Another key lipid, PS (O-17:0/22:6), exhibited excellent diagnostic performance for HD and PD, with AUCs of 1.000 and 0.9167, respectively, while showing moderate discrimination for AD (AUC = 0.5156). Consistently, PS (O-17:0/22:6) levels were significantly elevated in HD and PD, but unchanged in AD, relative to HV (Figure 3Bii). Similarly, PI (18:1/18:1) demonstrated strong discriminatory power with AUCs of 0.9722 and 0.9236 for HD and PD, and moderate differentiation for AD (AUC = 0.6736). The violin plot revealed significantly reduced PI (18:1/18:1) levels across all three disease groups compared with HV (Figure 3Biii). Collectively, these findings indicate that PS (16:1/24:0;O1), PS (O-17:0/22:6), and PI (18:1/18:1) may represent promising disease-specific lipid biomarkers for the diagnosis of AD, HD, and PD in older adults.

### 3.3 Region-specific brain lipidome alterations across AD, PD, and HD

**Figure 4:**
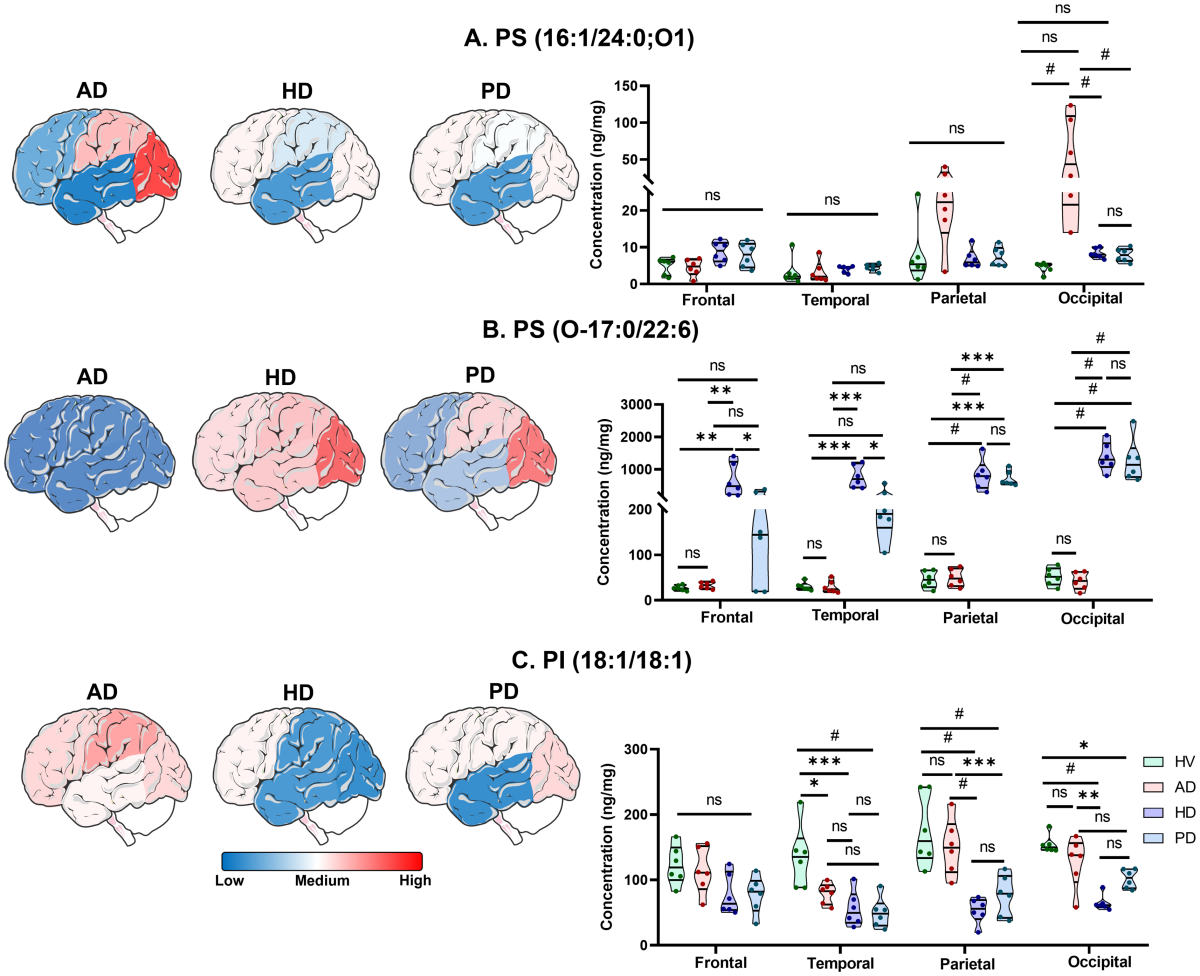
Brain-region heatmaps and violin plots illustrating region-specific distribution of significantly altered lipid species across AD, HD, and PD groups: **(A)** PS (16:1/24:0;O1), **(B)** PS (O-17:0/22:6), **(C)** PI (18:1/18:1). [Ordinary two-way ANOVA with Bonferroni’s multiple comparisons test, (95% CI), ^#^*p* < 0.0001, ^***^p < 0.001, ^**^p < 0.01, ^*^p < 0.05, and 0.1(ns)]. All data are represented as mean ± SEM.

Region-specific spatial distribution of significantly altered lipid species across frontal, temporal, parietal, and occipital regions in HV and patients with AD, PD, and HD is shown in Figure 4. The results revealed both disease-specific and region-dependent alterations in the levels of PS (16:1/24:0;O1). This lipid was significantly elevated in the AD group, with the greatest increase observed in the occipital cortex (*p* < 0.0001) and a modest, non-significant rise in the parietal region, suggesting possible localized oxidation of PS (16:1/24:1) in the occipital and parietal regions of the AD brain. No significant changes were detected in the frontal and temporal regions of AD or in any brain regions of the HD and PD groups relative to HV. The spatial distribution of PS (16:1/24:0;O1) is illustrated in the heatmaps and violin plots in Figure 4A. A distinct pattern of increased PS (O-17:0/22:6) levels was observed predominantly in the HD and PD groups. In HD, the parietal and occipital cortices showed pronounced increases (*p* < 0.0001), followed by moderate elevation in the temporal cortex (*p* < 0.001) and mild but significant changes in the frontal region (*p* < 0.01). Similarly, the PD group exhibited a strong increase in the occipital (*p* < 0.0001) and parietal (*p* < 0.001) regions, with non-significant trends in the frontal and temporal cortices. In contrast, the AD group showed no significant regional variation in PS (O-17:0/22:6) abundance. The region-specific distribution of this lipid species is shown in Figure 4B. Analysis of PI (18:1/18:1) revealed a consistent decrease across all three disease groups compared with HV. In HD, PI (18:1/18:1) levels were markedly reduced in the parietal and occipital regions (*p* < 0.0001), moderately decreased in the temporal lobe (*p* < 0.001), and unchanged in the frontal cortex. In PD, significant reductions were observed in the temporal and parietal regions (*p* < 0.0001), accompanied by a mild decrease in the occipital cortex (*p* < 0.05) but a non-significant difference in the frontal region. The AD group displayed minor, non-significant decreases across all four brain regions. Region-specific heatmaps and violin plots illustrating the distribution of PI (18:1/18:1) are presented in Figure 4C. Comprehensive violin plots summarizing disease- and region-specific distributions of total PS, oxidized PS (OxPS), ether-linked PS, and PI levels, along with a heatmap of the top 50 region-specific lipid species across HV, AD, HD, and PD groups, are provided in Supplementary Figures S1, S2.

### 3.4 Sex-specific brain lipid fingerprinting across AD, PD, and HD

**Figure 5:**
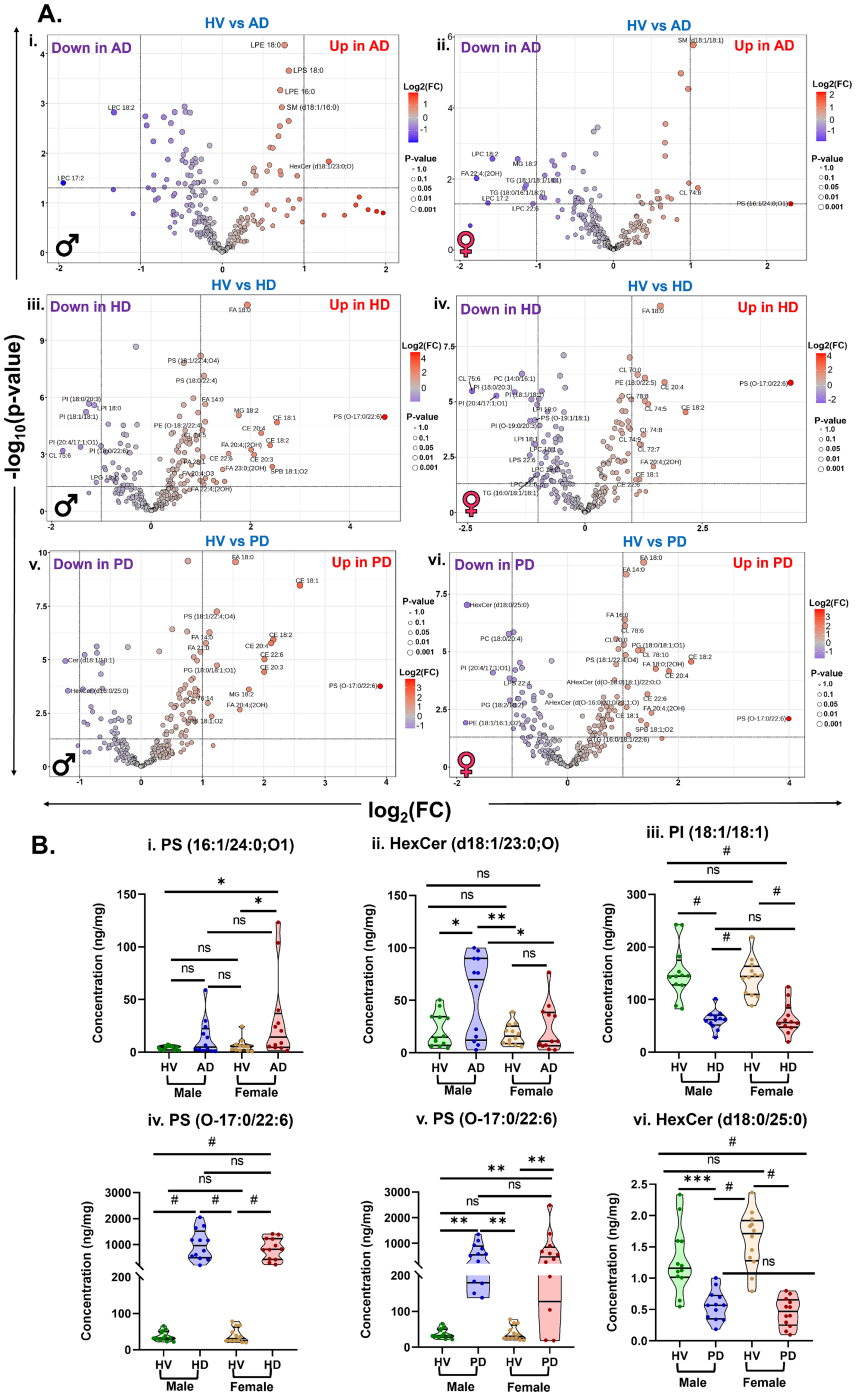
**(A)** Volcanic plot representing sex-specific significantly altered lipid levels (unpaired *t*-test, *p* < 0.05) in the brain samples comparing **(i,ii)** HV vs. AD, **(iii,iv)** HV vs. HD, and **(v,vi)** HV vs. PD from male and female participants. **(B)** Violin plots representing significantly altered lipids in the male and female participants of **(i,ii)** HV vs. AD, **(iii,iv)** HV vs. HD, and **(v,vi)** HV vs. PD. Sample sizes: HV: (12 males and 12 females), AD: (12 males and 12 females), HD: (12 males and 12 females), and PD: (12 males and 12 females). [Ordinary two-way ANOVA with Bonferroni’s multiple comparison test, (95% CI), ^#^*p* < 0.0001, ^***^p < 0.001, ^**^p < 0.01, ^*^p < 0.05, and 0.1(ns)]. All data are represented as mean ± SEM.

Sex-dependent variations in brain lipidomic alterations among HV, AD, PD, and HD are presented in Figure 5. Volcano plots illustrate key sex-specific lipid changes by plotting −log_10_(*p*-value) against log_2_(fold change), highlighting lipids with significant sex differences in abundance (Figure 5A). In the comparison between HV and AD groups, male participants exhibited increased levels of LPE 18:0, LPS 18:0, LPE 16:0, SM (d18:1/16:0), HexCer (d18:1/23:0;O), and decreased levels of LPC 17:2 and LPC 18:2 relative to HV (Figure 5Ai). In contrast, female AD participants displayed elevated levels of PS (16:1/24:0;O1), SM (d18:1/18:1), and CL 74:8, along with reduced concentrations of several LPCs and other lipids, including FA 22:4;(2OH), TG (18:1/18:1/18:2), and TG (18:0/16:1/18:2), compared with HV (Figure 5Aii). Among HD participants, males showed increased levels of multiple CEs, CLs, FAs, and PS (O-17:0/22:6), while several PI species, as well as LPG 18:1 and LPI 18:0, were decreased relative to HV (Figure 5Aiii). Female HD participants exhibited a comparable lipidome profile, characterized by increased levels of CEs, CLs, and FAs, accompanied by elevated PS (O-17:0/22:6) and reduced levels of PI species, including PI (18:1/18:1), PI (18:0/20:3), PI (20:4/17:1;O1), and PI (O-19:0/20:3), compared to HV (Figure 5Aiv).

Similarly, in the PD group, male participants displayed increased levels of PS (O-17:0/22:6), CEs, and FAs, while Cer (d18:1/18:1) and HexCer (d18:0/25:0) were decreased relative to HV (Figure 5Av). Female PD participants exhibited a similar trend, with elevated PS (O-17:0/22:6), FAs, CEs, and other lipid species, alongside decreased levels of HexCer (d18:0/25:0), PC (18:0/20:4), and other lipids compared with HV (Figure 5Avi). To further characterize these sex-related differences, violin plots were generated to visualize relative lipid abundances across male and female participants within each group (Figure 5B). In the AD cohort, PS (16:1/24:0;O1) was significantly elevated in females compared with HV, while no significant difference was observed in males (Figure 5Bi). Conversely, HexCer (d18:1/23:0;O) was significantly increased in male AD participants but remained unchanged in females (Figure 5Bii). In the HD group, PI (18:1/18:1) was significantly decreased in both sexes, whereas PS (O-17:0/22:6) was increased in both male and female participants relative to HV (Figures 5Biii,iv). Similarly, in PD, PS (O-17:0/22:6) levels were significantly elevated across both sexes, while HexCer (d18:0/25:0) concentrations were significantly reduced compared with HV (Figures 5Bv,vi).

## 4 Discussion

**Figure 6:**
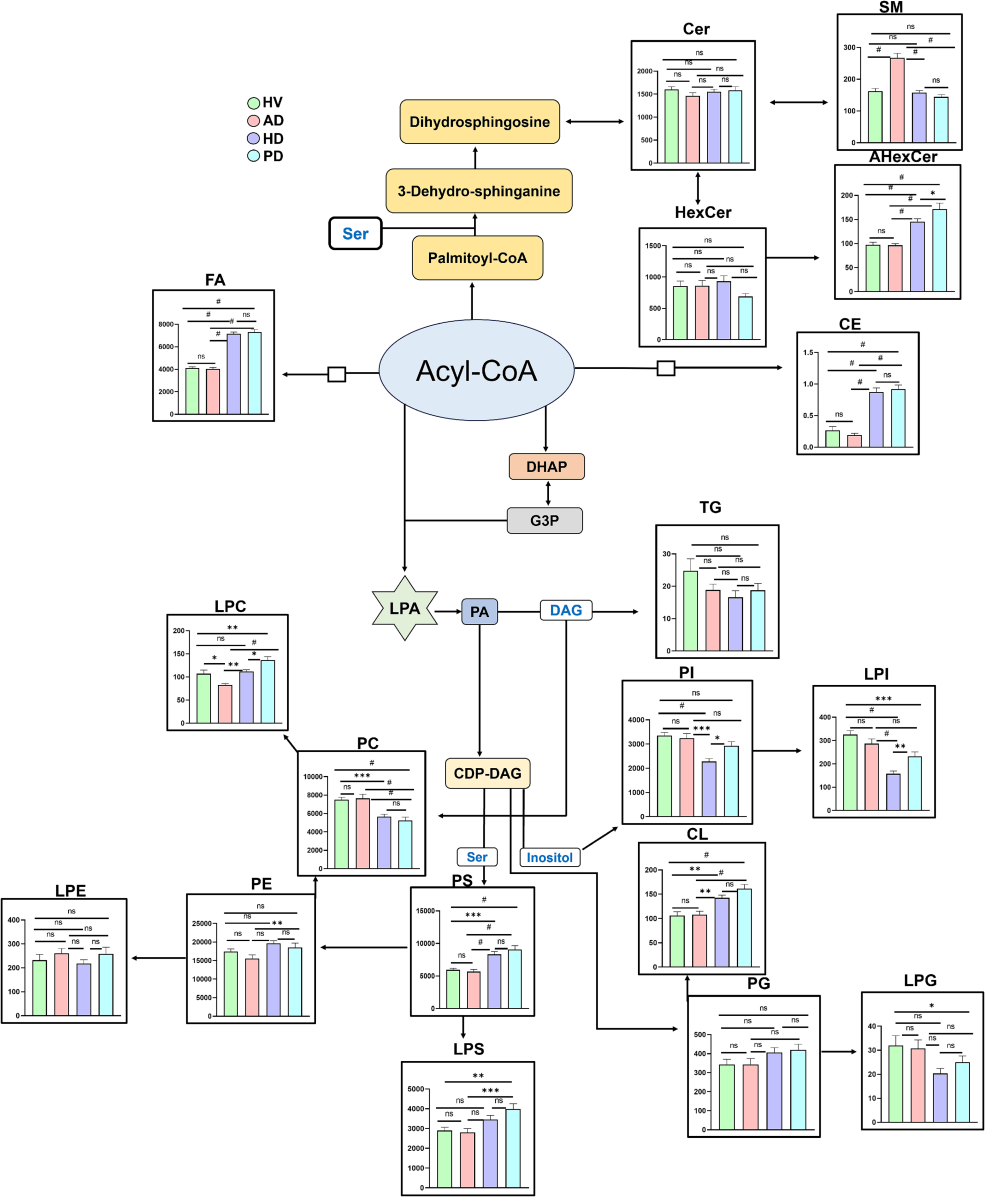
Lipid mapping of brain lipidome alterations in HV, AD, HD, and PD groups using biosynthesis pathway analysis. Data are shown as mean ± SEM (*y*-axis: lipid subclass concentration in ng/mg; *x*-axis: HV vs. AD, HD, and PD). Acetyl CoA, acetyl coenzyme A; FAs, fatty acids; DHAP, dihydroxyacetone phosphate; G3P, glycerol-3-phosphate; LPA, lysophosphatidic acid; PA, phosphatidic acid; CDP-DAG, cytidine diphosphate diacylglycerol; PI, phosphatidylinositol; PE, phosphatidylethanolamine; LPE, lyso-phosphatidylethanolamine; PC, phosphatidylcholine; LPC, lyso-phosphatidylcholine; PS, phosphatidylserine; DAG, diacylglycerol; TG, triacylglycerol; Ser, serine; Cer, ceramide; HexCer, hexosylceramide; AHexCer, acylhexosylceramide; SM, sphingomyelin. [Ordinary two-way ANOVA with Bonferroni’s multiple comparison test, (95% CI), ^#^*p* < 0.0001, ^***^p < 0.001, ^**^p < 0.01, ^*^p < 0.05, and 0.1(ns)].

To the best of our knowledge, this study represents the first comprehensive, untargeted brain lipidomic profiling of AD, HD, and PD patients, aimed at identifying potential disease-, region-, and sex-specific biomarkers for NDs diagnosis. Previous studies have shown that as the disease progresses, it will predominantly affect and widespread over other subcortical brain regions in the advanced stage of the disease (Brettschneider et al., 2015; Rowley et al., 2018; Holmes et al., 2024). In this study, we have obtained brain tissues from the patients at the advanced (dementia) stage of AD, PD, and HD, where pathology may extensively spread from subcortical origins into cortical regions across all four lobes: frontal, temporal, parietal, and occipital. Therefore, selecting these four cortical regions for lipidomic profiling is highly relevant for the discovery of disease biomarkers. The lipidomic alterations identified were contextualized using Kyoto Encyclopedia of Genes and Genomes (KEGG) lipid biosynthesis pathway^1^ analysis (Figure 6) (Jayaprakash et al., 2025). Multivariate analysis (Figures 2B–D) revealed clear separations between HV and each disease group, indicating substantial remodeling of lipid metabolism in these neurodegenerative disorders (Yang et al., 2022). The highest explained variance was observed in PD (43%), suggesting pronounced lipidomic heterogeneity, likely due to the selective vulnerability of dopaminergic neurons and mitochondrial lipid dysfunction (Tkachenko et al., 2025). FAs are essential for brain development, neurogenesis, and neurotransmitter synthesis (Youdim et al., 2000). In this study, FAs levels were significantly elevated in both HD and PD groups (Figure 6), consistent with previous reports describing increased FAs in HD plasma and PD CSF samples (McGarry et al., 2020; Fernández-Irigoyen et al., 2021). Specifically, elevated stearic acid (FA 18:0) in HD and PD brains (Figure 3A) may reflect reduced n9-desaturase activity, leading to impaired membrane fluidity and disrupted neuronal signaling, particularly within the frontal cortex of PD brains (Fabelo et al., 2011). Furthermore, saturated long-chain fatty acids, including FA 18:0 and myristic acid (FA 14:0) were increased in both the sex groups of HD and PD brains (Figure 5), indicating *de novo* fatty acid synthesis is closely associated with inflammatory cell subsets, which drive inflammation in both the sex groups of HD and PD (Bogie et al., 2020).

GPs are critical regulators of neuronal membrane stability and cell proliferation (Nitsch et al., 1991). Most GPs belonging subclass of lipids exhibited distinct alterations across diseases (Figure 6). LPC levels were significantly decreased in AD but increased in PD and HD. These results align with earlier findings of reduced LPC in AD brain tissues-implicated in neurotoxicity and inflammation (Kalia et al., 2023), and elevated LPC in the PD putamen, likely linked to altered phospholipase A2 activity under neuroinflammatory conditions (Fanning et al., 2019; Wu et al., 2021). Decrease in LPC levels in AD brains has previously been correlated to amyloid β load (Grimm et al., 2011).

Our study further found that the observed decrease in LPC levels was predominated by females (Figure 5A). These results are consistent with previous findings on decreased LPC in 3xTg-AD female mouse brain, indicating an increase in lysophospholipid acyltransferase activity (Strefeler et al., 2023).

PS lipids also participate in numerous neuronal signaling pathways (Ma et al., 2022). Elevated PS levels in PD frontal cortex promote α-synuclein aggregation on phospholipid membranes, driving neuronal degeneration (Fabelo et al., 2011; Canerina-Amaro et al., 2019). This pattern was reflected in our PD and HD results (Figure 6).

Oxidized forms of PS (OxPS), such as PS (16:0/24:1;O1), were elevated in the occipital and parietal cortices of AD brains, while ether-linked PS species [PS (O-17:0/22:6)] were increased in the temporal, parietal, and occipital regions of HD and PD brains (Figures 4A,B). These region-specific changes suggest ongoing neuroinflammatory processes and support their potential role as spatially defined biomarkers (Santos et al., 2024). Iron accumulation and lipid peroxidation are associated with the development of neurological disorders through the cystine/glutamate antiporter (System X_c_^−^), glutathione peroxidase 4 (GPX4), and glutathione (GSH) (System X_c_^−^/GSH/GPX4 axis) (Li et al., 2020). Elevated lipid oxidation serves as a key driver of ferroptosis in AD brain tissues-an iron-dependent form of regulated cell death characterized by the accumulation of phospholipid hydroperoxides (O’Donnell and Bochkov, 2025). This process leads to the functional impairment of GPX4, the primary enzyme responsible for reducing oxidized lipids via GPH (Seiler et al., 2008). A recent postmortem study reported reduced GPX4 expression levels in the vicinity of amyloid plaques in AD brains (Majerníková et al., 2024). In parallel, mitochondrial dysfunction (including small mitochondria with increased mitochondrial membrane densities, vanishing mitochondria Crista, and outer mitochondria memberane rupture) has been increasingly recognized as a crucial contributor to AD pathogenesis, exacerbating synaptic deficits, adenosine triphosphate (ATP) depletion, and oxidative stress through enhanced lipid peroxidation (Misrani et al., 2021; McGill Percy et al., 2025). Elevated reactive oxygen species (ROS) generation in the aging brain further intensifies oxidative lipid damage in AD (Alkhalifa et al., 2025). Additionally, increased ROS levels and ATP depletion have been observed in the plasma of AD patients, reflecting systemic oxidative imbalance (Ademowo et al., 2020). Collectively, these findings suggest that the observed increase in PS (16:0/24:1;O1) levels may result from GPX4 deficiency and excessive ROS production, particularly in females and within the occipital region of the AD brain.

The biological function of ether-PS species in HD and PD remains poorly understood and warrants further investigation. PIs are crucial regulators of neuronal cell signaling (Chun and Chung, 2020). Previous studies have documented decreased PI levels in the cerebral cortex of animal models of AD, PD, and HD (Waugh, 2015), which is consistent with the reductions observed here (Figure 6). This may indicate disrupted phosphoinositide signaling and its associated phosphatidylinositol phosphates biosynthesis, which are essential lipids for neuronal function (Chun and Chung, 2020). Specifically, PI (18:1/18:1) is recognized as a protective lipokine that supports neuronal survival. Its consistent decline across all brain regions in AD, PD, and HD (Figure 4C) suggests loss of neuroprotective capacity and increased susceptibility to oxidative stress and programmed cell death (Thürmer et al., 2022), reinforcing its value as a potential biomarker. Furthermore, PI(18:1/18:1) levels showed a significant decrease in HD brains across both sexes (Figure 5B), indicating that dysregulation of PI metabolism may contribute to synaptic plasticity impairment during HD progression (Farzana et al., 2023).

SPs are integral to neuronal membrane composition and intercellular signaling (Crivelli et al., 2020). Dysregulation of SPs metabolism has been implicated in the pathogenesis of multiple NDs (Hussain et al., 2019). Previous studies reported elevated sphingomyelin (SM) levels in the CSF and hippocampus of AD patients (Kosicek et al., 2012; Hsiao et al., 2013), in agreement with our findings (Figure 6). Further, these levels were increased in both the sex groups of AD patients (Figure 5). Increased SM may indicate overexpression of sphingomyelin synthase 1, which correlates with Aβ accumulation (Hsiao et al., 2013). Conversely, the observed reductions in Cer and HexCer species, particularly in male PD brains (Figure 5), may arise from downregulation of ceramide synthase-1, which contributes to PD pathogenesis (Abbott et al., 2014). Further, CEs play key roles in amyloidogenesis (Di Paolo and Kim, 2011), and excessive accumulation of CEs has been epidemiologically associated with increased dementia risk (Feringa and van der Kant, 2021). In this study, ω-6 fatty acid esterified CE accumulation was evident in HD and PD brain tissues (Figure 6), consistent with previous reports in the caudate and putamen regions of the HD brain (Phillips et al., 2020), suggesting upregulation of acyl-coenzyme A: cholesterol acyltransferase 1 may correlate with increased CEs in ND patients (Kim et al., 2011). The observed increase in CEs levels was evident in both the sex groups of HD and PD brain tissues; however, the exact sex-specific CE metabolism still needs further research (Figure 5).

Overall, this study provides novel insights into brain lipidome profiles across AD, PD, and HD, identifying 243 lipid molecular species. Multivariate analyses revealed distinct group separations between HV and disease groups. ROC analysis further identified PS (16:1/24:0;O1), PS (O-17:0/22:6), and PI (18:1/18:1) as promising diagnostic biomarker candidates for AD, HD, and PD, respectively. Regional analysis indicated that the occipital and parietal cortices are most affected across these disorders. Despite these advances, several limitations should be acknowledged. First, the study relied on semi-quantitative lipidomic data. Second, the relatively small sample size limits the statistical robustness of the specific findings. Third, the lack of control for potential major confounding factors such as diet, physical activity, chronic medication history, genetic background, and post-mortem interval (PMI) may influence the observed lipidome changes. ROC analysis was conducted using the discovery set; however, the absence of replication and validation in independent cohorts represents a key limitation. Validation in an external cohort, ideally using plasma or CSF samples from living patients, is essential to confirm the reproducibility of the present findings and to establish their potential clinical utility. This study is limited to only four cortical regions across control and AD, HD, and PD individuals. Future studies should incorporate primary disease epicenters to delineate lipidomic alterations during early pathological stages in disease progression. There is a general acceptance that aberrant misfolding and non-physiological aggregation of Aβ in AD, α- synuclein in PD, and huntingtin protein in HD damage the brain and eventually lead to neuronal loss. As a result, the overall observed lipid alterations may reflect both intrinsic metabolic reprogramming and secondary changes in cellular composition (e.g., neuronal loss, gliosis), which warrants future cellular lipidomic studies to validate the identified lipid biomarkers. Mechanistic linkages between the observed lipid dysregulation and neurodegenerative pathology remain to be fully elucidated.

In summary, this study constitutes the first comparative lipidomic investigation of AD, HD, and PD brain tissues using untargeted lipidomics, revealing disease-specific lipid alterations and identifying PS (16:1/24:0;O1), PS (O-17:0/22:6), and PI (18:1/18:1) as potential lipid biomarkers. These findings advance our understanding of lipid metabolism in neurodegeneration and underscore the need for future mechanistic and longitudinal studies to validate these biomarkers and clarify their roles in disease progression.

## Data Availability

The datasets presented in this study can be found in online repositories. The names of the repository/repositories and accession number(s) can be found in the article/Supplementary material.

## Footnotes

1
https://www.genome.jp/kegg/pathway.html

## Supplementary material

SUPPLEMENTARY TABLE S1 Relative concentration (ng/mg) of quantified lipids in brain samples.

SUPPLEMENTARY TABLE S2 Top altered lipid molecular species between HV and AD groups of brain tissue samples (Unpaired t-test, *p* <0.05).

SUPPLEMENTARY TABLE S3 Top altered lipid molecular species between HV and HD groups of brain tissue samples (Unpaired t-test, *p* <0.05).

SUPPLEMENTARY TABLE S4 Top altered lipid molecular species between HV and PD groups of brain tissue samples (Unpaired t-test, *p* <0.05).

SUPPLEMENTARY FIGURE S1 Violin plots of disease-specific and region-specific alterations in lipid classes between HV and disease groups (AD, HD, and PD) in A. Total PS, B. Total oxidized PS, C. Total ether-PS, and D. Total PI. (Ordinary two-way ANOVA with Bonferroni’s multiple comparison test, (95% CI), #*p*< 0.0001, ****p*<0.001, ***p*<0.01, **p*<0.05, 0.1(ns)). Data = mean ± SEM.

SUPPLEMENTARY FIGURE S2 Heatmaps of the top 50 altered lipids across frontal (F), temporal (T), parietal (P), and occipital (O) regions of A. HV and AD, B. HV and HD, and C. HV and PD. Red = higher concentration; blue = lower concentration. (Abbreviation: HV: Healthy volunteers, AD: Alzheimer’s Disease, HD: Huntington’s Disease, and PD: Parkinson’s Disease).

## References

[ref1] Abbott S. K. Li H. Muñoz S. S. Knoch B. Batterham M. Murphy K. E. . (2014). Altered ceramide acyl chain length and ceramide synthase gene expression in Parkinson’s disease. Mov. Disord. 29, 518–526. doi: 10.1002/mds.25729, 24822250

[ref2] Ademowo O. S. Dias I. H. K. Diaz-Sanchez L. Sanchez-Aranguren L. Stahl W. Griffiths H. R. (2020). Partial mitigation of oxidized phospholipid-mediated mitochondrial dysfunction in neuronal cells by oxocarotenoids. J. Alzheimers Dis. 74, 113–126. doi: 10.3233/JAD-190923, 31985464

[ref3] Alkhalifa A. Alkhalifa O. Durdanovic I. Ibrahim D. R. Maragkou S. (2025). Oxidative stress and mitochondrial dysfunction in Alzheimer’s disease: insights into pathophysiology and treatment. J. Dement. Alzheimers Dis. 2:17. doi: 10.3390/jdad2020017

[ref4] Bloom G. S. (2014). Amyloid-β and tau: the trigger and bullet in Alzheimer disease pathogenesis. JAMA Neurol. 71, 505–508. doi: 10.1001/jamaneurol.2013.5847, 24493463 PMC12908160

[ref5] Bogie J. F. J. Haidar M. Kooij G. Hendriks J. J. A. (2020). Fatty acid metabolism in the progression and resolution of CNS disorders. Adv. Drug Deliv. Rev. 159, 198–213. doi: 10.1016/j.addr.2020.01.004, 31987838

[ref6] Brettschneider J. Del Tredici K. Lee V. M.-Y. Trojanowski J. Q. (2015). Spreading of pathology in neurodegenerative diseases: a focus on human studies. Nat. Rev. Neurosci. 16, 109–120. doi: 10.1038/nrn3887, 25588378 PMC4312418

[ref7] Byeon S. K. Madugundu A. K. Jain A. P. Bhat F. A. Jung J. H. Renuse S. . (2021). Cerebrospinal fluid lipidomics for biomarkers of Alzheimer’s disease. Mol. Omics 17, 454–463. doi: 10.1039/d0mo00186d, 34125126 PMC8210464

[ref8] Canerina-Amaro A. Pereda D. Diaz M. Rodriguez-Barreto D. Casañas-Sánchez V. Heffer M. . (2019). Differential aggregation and phosphorylation of alpha synuclein in membrane compartments associated with Parkinson disease. Front. Neurosci. 13:382. doi: 10.3389/fnins.2019.00382, 31068782 PMC6491821

[ref9] Chun Y. S. Chung S. (2020). High-cholesterol diet decreases the level of phosphatidylinositol 4,5-bisphosphate by enhancing the expression of phospholipase C (PLCβ1) in rat brain. Int. J. Mol. Sci. 21:1161. doi: 10.3390/ijms21031161, 32050555 PMC7038105

[ref10] Crivelli S. M. Giovagnoni C. Visseren L. Scheithauer A.-L. de Wit N. den Hoedt S. . (2020). Sphingolipids in Alzheimer’s disease, how can we target them? Adv. Drug Deliv. Rev. 159, 214–231. doi: 10.1016/j.addr.2019.12.003, 31911096

[ref11] Di Paolo G. Kim T. W. (2011). Linking lipids to Alzheimer’s disease: cholesterol and beyond. Nat. Rev. Neurosci. 12, 284–296. doi: 10.1038/nrn3012, 21448224 PMC3321383

[ref12] Fabelo N. Martín V. Santpere G. Marín R. Torrent L. Ferrer I. . (2011). Severe alterations in lipid composition of frontal cortex lipid rafts from Parkinson’s disease and incidental Parkinson’s disease. Mol. Med. 17, 1107–1118. doi: 10.2119/molmed.2011.00119, 21717034 PMC3188884

[ref13] Fanning S. Haque A. Imberdis T. Baru V. Barrasa M. I. Nuber S. . (2019). Lipidomic analysis of α-synuclein neurotoxicity identifies stearoyl CoA desaturase as a target for Parkinson treatment. Mol. Cell 73, 1001–1014.e8. doi: 10.1016/j.molcel.2018.11.028, 30527540 PMC6408259

[ref14] Farzana F. McConville M. J. Renoir T. Li S. Nie S. Tran H. . (2023). Longitudinal spatial mapping of lipid metabolites reveals pre-symptomatic changes in the hippocampi of Huntington’s disease transgenic mice. Neurobiol. Dis. 176:105933. doi: 10.1016/j.nbd.2022.105933, 36436748

[ref15] Feringa F. M. den Koppes- Hertog S. J. Wang L. Y. Derks R. J. E. Kruijff I. Erlebach L. . (2025). The neurolipid atlas: a lipidomics resource for neurodegenerative diseases. Nat. Metab. 7, 2142–2164. doi: 10.1038/s42255-025-01365-z, 40983680 PMC12552125

[ref16] Feringa F. M. van der Kant R. (2021). Cholesterol and Alzheimer’s disease; from risk genes to pathological effects. Front. Aging Neurosci. 13:690372. doi: 10.3389/fnagi.2021.690372, 34248607 PMC8264368

[ref17] Fernández-Irigoyen J. Cartas-Cejudo P. Iruarrizaga-Lejarreta M. Santamaría E. (2021). Alteration in the cerebrospinal fluid lipidome in Parkinson’s disease: a post-mortem pilot study. Biomedicine 9:491. doi: 10.3390/biomedicines9050491, 33946950 PMC8146703

[ref18] Folch J. Lees M. Sloane Stanley G. H. (1957). A simple method for the isolation and purification of total lipides from animal tissues. J. Biol. Chem. 226, 497–509. doi: 10.1016/s0021-9258(18)64849-5, 13428781

[ref19] GBD 2016 Parkinson’s Disease Collaborators (2018). Global, regional, and national burden of Parkinson’s disease, 1990–2016: a systematic analysis for the Global Burden of Disease Study 2016. Lancet Neurol. 17, 939–953. doi: 10.1016/S1474-4422(18)30295-3, 30287051 PMC6191528

[ref20] GBD 2019 Dementia Forecasting Collaborators (2022). Estimation of the global prevalence of dementia in 2019 and forecasted prevalence in 2050: an analysis for the Global Burden of Disease Study 2019. Lancet Public Health 7, e105–e125. doi: 10.1016/S2468-2667(21)00249-8, 34998485 PMC8810394

[ref21] Gizaw S. T. (2015). “Comprehensive glycomics for the discovery of new biomarkers in neurodegenerative diseases” in Doctoral Dissertation (Sapporo: Hokkaido University Graduate School of Life Sciences).

[ref22] Grimm M. O. W. Grösgen S. Riemenschneider M. Tanila H. Grimm H. S. Hartmann T. (2011). From brain to food: analysis of phosphatidylcholins, lyso-phosphatidylcholins and phosphatidylcholin-plasmalogens derivates in Alzheimer’s disease human post mortem brains and mice model via mass spectrometry. J. Chromatogr. A 1218, 7713–7722. doi: 10.1016/j.chroma.2011.07.073, 21872257

[ref23] Hansson O. (2021). Biomarkers for neurodegenerative diseases. Nat. Med. 27, 954–963. doi: 10.1038/s41591-021-01382-x, 34083813

[ref24] Holmes S. E. Honhar P. Tinaz S. Naganawa M. Hilmer A. T. Gallezot J.-D. . (2024). Synaptic loss and its association with symptom severity in Parkinson’s disease. npj Parkinsons Dis. 10:42. doi: 10.1038/s41531-024-00655-9, 38402233 PMC10894197

[ref25] Hornemann T. (2021). Mini review: lipids in peripheral nerve disorders. Neurosci. Lett. 740:135455. doi: 10.1016/j.neulet.2020.135455, 33166639

[ref26] Hsiao J.-H. T. Fu Y. Hill A. F. Halliday G. M. Kim W. S. (2013). Elevation in sphingomyelin synthase activity is associated with increases in amyloid-beta peptide generation. PLoS One 8:e74016. doi: 10.1371/journal.pone.0074016, 23977395 PMC3748018

[ref27] Hussain G. Wang J. Rasul A. Anwar H. Imran A. Qasim M. . (2019). Role of cholesterol and sphingolipids in brain development and neurological diseases. Lipids Health Dis. 18:26. doi: 10.1186/s12944-019-0965-z, 30683111 PMC6347843

[ref28] Jayaprakash J. Gowda S. G. B. Gowda D. Ikeda A. Bamai Y. A. Ketema R. M. . (2025). Plasma lipidomics of preadolescent children: a Hokkaido study. J. Lipids 2025:3106145. doi: 10.1155/jl/3106145, 40084067 PMC11898111

[ref29] Kalia V. Reyes-Dumeyer D. Dubey S. Nandakumar R. Lee A. J. Lantigua R. . (2023). Lysophosphatidylcholines are associated with P-tau181 levels in early stages of Alzheimer’s disease. *medRxiv*. Available online at: 10.1101/2023.08.24.23294581. [Epub ahead of preprint] PMC12823400 41407938

[ref30] Katsuno M. Sahashi K. Iguchi Y. Hashizume A. (2018). Preclinical progression of neurodegenerative diseases. Nagoya J. Med. Sci. 80, 289–298. doi: 10.18999/nagjms.80.3.289, 30214078 PMC6125655

[ref31] Khoury R. Ghossoub E. (2019). Diagnostic biomarkers of Alzheimer’s disease: a state-of-the-art review. Biomark. Neuropsychiatry 1:100005. doi: 10.1016/j.bionps.2019.100005

[ref32] Kim J.-H. Ee S.-M. Jittiwat J. Ong E.-S. Farooqui A. A. Jenner A. M. . (2011). Increased expression of acyl-coenzyme a: cholesterol acyltransferase-1 and elevated cholesteryl esters in the hippocampus after excitotoxic injury. Neuroscience 185, 125–134. doi: 10.1016/j.neuroscience.2011.04.018, 21514367

[ref33] Kosicek M. Zetterberg H. Andreasen N. Peter-Katalinic J. Hecimovic S. (2012). Elevated cerebrospinal fluid sphingomyelin levels in prodromal Alzheimer’s disease. Neurosci. Lett. 516, 302–305. doi: 10.1016/j.neulet.2012.04.019, 22521584

[ref34] Lamptey R. N. L. Chaulagain B. Trivedi R. Gothwal A. Layek B. Singh J. (2022). A review of the common neurodegenerative disorders: current therapeutic approaches and the potential role of nanotherapeutics. Int. J. Mol. Sci. 23:1851. doi: 10.3390/ijms23031851, 35163773 PMC8837071

[ref35] Li J. Cao F. Yin H.-L. Huang Z.-J. Lin Z.-T. Mao N. . (2020). Ferroptosis: past, present and future. Cell Death Dis. 11:88. doi: 10.1038/s41419-020-2298-2, 32015325 PMC6997353

[ref36] Li T. Le W. (2020). Biomarkers for Parkinson’s disease: how good are they? Neurosci. Bull. 36, 183–194. doi: 10.1007/s12264-019-00433-1, 31646434 PMC6977795

[ref37] Liu Y. Thalamuthu A. Mather K. A. Crawford J. Ulanova M. Wong M. W. K. . (2021). Plasma lipidome is dysregulated in Alzheimer’s disease and is associated with disease risk genes. Transl. Psychiatry 11:344. doi: 10.1038/s41398-021-01362-2, 34092785 PMC8180517

[ref38] Ma X. Li X. Wang W. Zhang M. Yang B. Miao Z. (2022). Phosphatidylserine, inflammation, and central nervous system diseases. Front. Aging Neurosci. 14:975176. doi: 10.3389/fnagi.2022.975176, 35992593 PMC9382310

[ref39] Majerníková N. Marmolejo-Garza A. Salinas C. S. Luu M. D. A. Zhang Y. Trombetta-Lima M. . (2024). The link between amyloid β and ferroptosis pathway in Alzheimer’s disease progression. Cell Death Dis. 15:782. doi: 10.1038/s41419-024-07152-0, 39468028 PMC11519607

[ref40] Martens G. A. Geßner C. Folkow L. P. Creydt M. Fischer M. Burmester T. (2023). The roles of brain lipids and polar metabolites in the hypoxia tolerance of deep-diving pinnipeds. J. Exp. Biol. 226:jeb245355. doi: 10.1242/jeb.245355, 36970764

[ref41] McGarry A. Gaughan J. Hackmyer C. Lovett J. Khadeer M. Shaikh H. . (2020). Cross-sectional analysis of plasma and CSF metabolomic markers in Huntington’s disease for participants of varying functional disability: a pilot study. Sci. Rep. 10:20490. doi: 10.1038/s41598-020-77526-9, 33235276 PMC7686309

[ref42] McGill Percy K. C. Liu Z. Qi X. (2025). Mitochondrial dysfunction in Alzheimer’s disease: guiding the path to targeted therapies. Neurotherapeutics 22:e00525. doi: 10.1016/j.neurot.2025.e00525, 39827052 PMC12047401

[ref43] Minami Y. Gowda S. G. B. Gowda D. Chiba H. Hui S. -P. (2025). Sex- and Regio-Specific Lipid Profiling of Shishamo and Capelin Fish by Nontargeted Liquid Chromatography/Mass Spectrometry. Foods. 15:298. doi: 10.3390/foods15020298 PMC12840183 41596897

[ref44] Misrani A. Tabassum S. Yang L. (2021). Mitochondrial dysfunction and oxidative stress in Alzheimer’s disease. Front. Aging Neurosci. 13:617588. doi: 10.3389/fnagi.2021.617588, 33679375 PMC7930231

[ref45] Nath L. R. Gowda S. G. B. Roberts T. H. Gowda D. Khoddami A. Hui S.-P. (2024). Nontargeted lipidomics of Sorghum grain reveals novel fatty acid esters of hydroxy fatty acids and cultivar differences in lipid profiles. J. Agric. Food Chem. 72, 20690–20703. doi: 10.1021/acs.jafc.4c05919, 39230960

[ref46] Nitsch R. Pittas A. Blusztajn J. K. Slack B. E. Growdon J. H. Wurtman R. J. (1991). Alterations of phospholipid metabolites in postmortem brain from patients with Alzheimer’s disease. Ann. N. Y. Acad. Sci. 640, 110–113. doi: 10.1111/j.1749-6632.1991.tb00200.x, 1663712

[ref47] O’Brien J. S. Sampson E. L. (1965). Lipid composition of the normal human brain: gray matter, white matter, and myelin. J. Lipid Res. 6, 537–544. 5865382

[ref48] O’Donnell V. B. Bochkov V. (2025). Oxidized phospholipids in ferroptosis, immunity and inflammation. Redox Biochem. Chem. 14:100061. doi: 10.1016/j.rbc.2025.100061

[ref49] Paryani F. Kwon J.-S. Ng C. W. Jakubiak K. Madden N. Ofori K. . (2024). Multi-omic analysis of Huntington’s disease reveals a compensatory astrocyte state. Nat. Commun. 15:6742. doi: 10.1038/s41467-024-50626-0, 39112488 PMC11306246

[ref50] Phillips G. R. Hancock S. E. Brown S. H. J. Jenner A. M. Kreilaus F. Newell K. A. . (2020). Cholesteryl ester levels are elevated in the caudate and putamen of Huntington’s disease patients. Sci. Rep. 10:20314. doi: 10.1038/s41598-020-76973-8, 33219259 PMC7680097

[ref51] Rissardo J. P. Gadelmawla A. F. Khalil I. Abdulgadir A. Bhatti K. S. Fornari Caprara A. L. (2025). Epidemiology of autonomic dysfunction in Parkinson’s disease (review). Med. Int. 5:68. doi: 10.3892/mi.2025.267, 41018272 PMC12464526

[ref52] Rowley C. D. Tabrizi S. J. Scahill R. I. Leavitt B. R. Roos R. A. C. Durr A. . (2018). Altered intracortical T1-weighted/T2-weighted ratio signal in Huntington’s disease. Front. Neurosci. 12:805. doi: 10.3389/fnins.2018.00805, 30455625 PMC6230564

[ref53] Sanni A. Bennett A. I. Adeniyi M. Mechref Y. (2025). Dysregulated lipids in Alzheimer’s disease: insights into biological pathways through LC-MS/MS analysis of human brain tissues. ACS Chem. Neurosci. 16, 3694–3712. doi: 10.1021/acschemneuro.5c00230, 40957103

[ref54] Santos M. Melo T. Maurício T. Ferreira H. Domingues P. Domingues R. (2024). The non-enzymatic oxidation of phosphatidylethanolamine and phosphatidylserine and their intriguing roles in inflammation dynamics and diseases. FEBS Lett. 598, 2174–2189. doi: 10.1002/1873-3468.14992, 39097985

[ref55] Seiler A. Schneider M. Förster H. Roth S. Wirth E. K. Culmsee C. . (2008). Glutathione peroxidase 4 senses and translates oxidative stress into 12/15-lipoxygenase dependent- and AIF-mediated cell death. Cell Metab. 8, 237–248. doi: 10.1016/j.cmet.2008.07.005, 18762024

[ref56] Selvam S. Ayyavoo V. (2024). Biomarkers in neurodegenerative diseases: a broad overview. Explor. Neuroprot. Ther. 4, 119–147. doi: 10.37349/ent.2024.00075

[ref57] Strefeler A. Jan M. Quadroni M. Teav T. Rosenberg N. Chatton J.-Y. . (2023). Molecular insights into sex-specific metabolic alterations in Alzheimer’s mouse brain using multi-omics approach. Alzheimers Res. Ther. 15:8. doi: 10.1186/s13195-023-01162-4, 36624525 PMC9827669

[ref58] Thürmer M. Gollowitzer A. Pein H. Neukirch K. Gelmez E. Waltl L. . (2022). PI(18:1/18:1) is a SCD1-derived lipokine that limits stress signaling. Nat. Commun. 13:2982. doi: 10.1038/s41467-022-30374-9, 35624087 PMC9142606

[ref59] Tkachenko K. González-Sáiz J. M. Pizarro C. (2025). Untargeted lipidomic reveals potential biomarkers in plasma samples for the discrimination of patients affected by Parkinson’s disease. Molecules 30:850. doi: 10.3390/molecules30040850, 40005161 PMC11857942

[ref60] Waugh M. G. (2015). PIPs in neurological diseases. Biochim. Biophys. Acta 1851, 1066–1082. doi: 10.1016/j.bbalip.2015.02.002, 25680866

[ref61] Wu Z. Wu S. Liang T. Wang L. (2021). Lipoprotein-associated phospholipase A2 is a risk factor for patients with Parkinson’s disease. Front. Neurosci. 15:633022. doi: 10.3389/fnins.2021.633022, 33958981 PMC8093434

[ref62] Yang D. Wang X. Zhang L. Fang Y. Zheng Q. Liu X. . (2022). Lipid metabolism and storage in neuroglia: role in brain development and neurodegenerative diseases. Cell Biosci. 12:106. doi: 10.1186/s13578-022-00828-0, 35831869 PMC9277953

[ref63] Yilmaz A. Akyol S. Ashrafi N. Saiyed N. Turkoglu O. Graham S. F. (2025a). Lipidomics of Huntington’s disease: a comprehensive review of current status and future directions. Metabolites 15:10. doi: 10.3390/metabo15010010, 39852353 PMC11766911

[ref64] Yilmaz A. Ashrafi N. Ashrafi R. Akyol S. Saiyed N. Kerševičiūtė I. . (2025b). Lipid profiling of Parkinson’s disease brain highlights disruption in lysophosphatidylcholines, and triacylglycerol metabolism. npj Parkinsons Dis. 11:159. doi: 10.1038/s41531-025-01023-x, 40500301 PMC12159154

[ref65] Yoon J. H. Seo Y. Jo Y. S. Lee S. Cho E. Cazenave-Gassiot A. . (2022). Brain lipidomics: from functional landscape to clinical significance. Sci. Adv. 8:eadc9317. doi: 10.1126/sciadv.adc9317, 36112688 PMC9481132

[ref66] Youdim K. A. Martin A. Joseph J. A. (2000). Essential fatty acids and the brain: possible health implications. Int. J. Dev. Neurosci. 18, 383–399. doi: 10.1016/s0736-5748(00)00013-7, 10817922

[ref67] Zhang X. Liu W. Zan J. Wu C. Tan W. (2020). Untargeted lipidomics reveals progression of early Alzheimer’s disease in APP/PS1 transgenic mice. Sci. Rep. 10:14509. doi: 10.1038/s41598-020-71510-z, 32884056 PMC7471266

